# Dental Practice Integration Into Primary Care: A Microsimulation of Financial Implications for Practices

**DOI:** 10.3390/ijerph17062154

**Published:** 2020-03-24

**Authors:** Sung Eun Choi, Lisa Simon, Jane R. Barrow, Nathan Palmer, Sanjay Basu, Russell S. Phillips

**Affiliations:** 1 Department of Oral Health Policy and Epidemiology, Harvard School of Dental Medicine, Boston, MA 02115, USA; 2 Office of Global and Community Health, Harvard School of Dental Medicine, Boston, MA 02115, USA; Lisa_Simon@hms.harvard.edu (L.S.); Jane_Barrow@hsdm.harvard.edu (J.R.B); 3 Harvard Medical School, Boston, MA 02115, USA; 4 Department of Biomedical Informatics, Harvard Medical School, Boston, MA 02115, USA; Nathan_Palmer@hms.harvard.edu; 5 Center for Primary Care, Harvard Medical School, Boston, MA 02115, USA; Sanjay_Basu@hms.harvard.edu (S.B.); Russell_Phillips@hms.harvard.edu (R.S.P.); 6 Research and Analytics, Collective Health, San Francisco, CA 94107, USA; 7 School of Public Health, Imperial College London, London SW7 2BU, UK

**Keywords:** integrated care, medical–dental integration, simulation model, dental research

## Abstract

Given the widespread lack of access to dental care for many vulnerable Americans, there is a growing realization that integrating dental and primary care may provide comprehensive care. We sought to model the financial impact of integrating dental care provision into a primary care practice. A microsimulation model was used to estimate changes in net revenue per practice by simulating patient visits to a primary dental practice within primary care practices, utilizing national survey and un-identified claims data from a nationwide health insurance plan. The impact of potential changes in utilization rates and payer distributions and hiring additional staff was also evaluated. When dental care services were provided in the primary care setting, annual net revenue changes per practice were −$92,053 (95% CI: −93,054, −91,052) in the first year and $104,626 (95% CI: 103,315, 105,316) in subsequent years. Net revenue per annum after the first year of integration remained positive as long as the overall utilization rates decreased by less than 25%. In settings with a high proportion of publicly insured patients, the net revenue change decreased but was still positive. Integrating primary dental and primary care providers would be financially viable, but this viability depends on demands of dental utilization and payer distributions.

## 1. Introduction

Dentistry has traditionally remained a separate discipline from other areas of medicine in the U.S. [1], and this artificial division does not foster comprehensive and high-quality care. Evidence shows that oral health complications, such as inflammation and infections that begin in the mouth, can lead to major health complications (e.g., dental abscess) [2]. Furthermore, a growing body of research has identified a potential connection between oral health and other chronic conditions, such as diabetes and cardiovascular diseases [3,4,5]. The National Academy of Medicine (an American nonprofit, non-governmental organization providing expert advice on issues relating to health, medicine, and health policy) has proposed integrating oral health into primary care as a way to expand access to recommended treatments and promote better health overall [6,7]. Despite recent studies suggesting that integration of dental care may benefit patients or reduce healthcare costs [8], financing and delivery of dental care remains disconnected from other health services, even among Accountable Care Organizations (ACOs), a network of coordinated healthcare practitioners in the U.S. that shares financial and medical responsibility for providing coordinated care to patients in the hopes of improving overall population health. Integration of dental care may present an opportunity for improved accountability for total health. However, there is little financial incentive and considerable financial uncertainties for ACOs to facilitate access to these services [6,9,10].

A number of organizations have initiated efforts to adopt integrated dental–medical care. One form of these efforts is integration in a co-located setting where provision of primary dental services is within and a part of primary care or vice versa. Co-location of medical and dental services is not a new concept; Federally Qualified Health Centers across the country have offered medical and dental facilities in the same building for decades, but often, electronic health records (EHRs) lack interoperability. A more innovative co-located model would allow communication across disciplines and sharing of patient information and EHRs, which provides an opportunity for the providers to “close the loop” on care gaps for patients beyond just providing care [11,12]. This approach facilitates timely delivery of diagnostic, preventive, and treatment services to improve patient health and reduce inefficiency in care delivery, allowing easier bidirectional referrals and quicker access for medical patients with acute oral health situations (and for dental patients with potential medical issues) [3,4,5,13].

Currently there are co-located facilities developing in the U.S., and pilot studies are being conducted in these settings [14]. A number of integrated care projects have had promising results, including the Colorado Medical Dental Integration Project [15,16]. One of the demonstration projects, the University of California, Los Angeles (UCLA)-First 5 LA Project, showed increased access to dental care by 85%, with the majority of services in diagnostic and preventive care [17]. While these demonstration projects are effective in assessing changes in dental care access rates and identifying logistical barriers, a key gap in knowledge is the economic viability of the delivery of such services by primary care practices constrained by financial realities. In this study, we estimated the cost and revenue implications to primary care practices of embedding a dental practice to integrate primary dental and primary medical care.

## 2. Materials and Methods

### 2.1. Study Design

**Figure 1:**
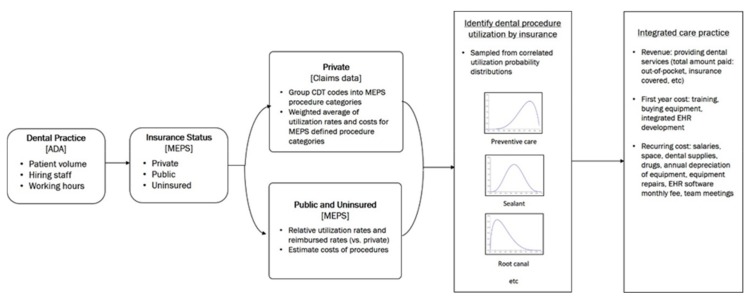
Simulation model flow diagram [data sources]. ADA = American Dental Association; MEPS = Medical Expenditure Panel Survey; CDT = Code on Dental Procedures and Nomenclature.

We estimated costs and revenues for an integrated medical and dental practice using a microsimulation model (Figure 1), an approach often used to evaluate the effects of hypothetical interventions before they are implemented in the real world [8,18]. We simulated a representative sample of 10,000 integrated practices (dental practice embedded within the primary care practice providing dental services provided by a general dentist and dental hygienist, with supporting dental assistants), per International Society for Pharmacoeconomics and Outcomes Research (ISPOR) guidelines [19]. For each of the simulated practices, we assigned a number of simulated patient visits, then for each visit, an insurance type and indicator variables for receiving certain types of procedures were assigned, matching the overall distribution of procedure utilization rates by insurance type. 

The simulation model was re-run 10,000 times while repeatedly Monte Carlo sampling from the probability distributions around the patient volume, utilization, cost, and expense data points shown in Table 1 to compute the mean and 95% credible intervals [20]. This process also accounted for the correlation among procedure utilization rates by insurance type to capture the common co-occurrence of procedures. Simulations were performed in R (v. 3.3.2, The R Foundation for Statistical Computing, Vienna, Austria). This study was reviewed by the institutional review board of the Harvard Medical School and determined to be “not-human subjects research” since the data are publicly available and de-identified.

### 2.2. Model Assumption

We first estimated the patient volume that needs to be maintained at the integrated settings. On average, full-time equivalent (FTE) general dental practitioners experience 14.6 patient visits per day including dental hygienist visits [21]. An FTE primary care physician sees 19.7 patients per day on average [30]. In our model, we assumed that the minimum patient volume at the integrated settings is at least 15 patients per day, the supply of dentists remains above 61 dentists per 100,000 population with 5 primary care physicians to 1 general dental practitioner per setting. Then, we identified dental procedures that could be routinely offered by general dentists using the Code on Dental Procedures and Nomenclature (CDT Code) [31]. The final set of procedures offered in the primary care setting was determined based on the list of dental procedures covered by Adult Medicaid dental benefits in Maryland and by expert opinions from more than two general dentists to determine a conservative set of procedures (Supplementary Table S4) [32]. This final set of procedures does not include procedures that involve cost-prohibitive dental equipment for a small general dental practice, such as a Panorex machine, or are primarily billed by dentist specialists, such as orthodontic services.

### 2.3. Data Sources

Data sources and input data for the model are detailed in Table 1. We obtained the annual patient volume and transition costs from American Dental Association (ADA) Survey of Dental Practice [21,33]. We then subcategorized dental visits for each procedure type among patients by dental insurance type: private, public, and self-pay/uninsured based on Medical Expenditure Panel Survey (MEPS) data (for dental practices; *N* = 30.5 million) (Figure 1) [22]. 

We obtained the utilization rates and costs for each procedure among a privately insured population using un-identifiable member claims data from Aetna and estimated utilization rates and cost (reimbursed rates and payer distribution) among publicly insured and uninsured populations by extrapolating from MEPS (Supplementary Tables S2 and S3 and Supplementary Figure S1) [22]. Because MEPS data do not provide procedure-level utilization rates, we grouped CDT procedure codes into the procedure categories used in MEPS (Supplementary Table S4). These estimates were used to capture varying utilization and reimbursement rates by insurance status across the U.S. 

### 2.4. Cost and Revenue Estimates from Dental–Medical Integration

We computed the cost of the embedded dental practice using procedure utilization rates and associated costs (shown in Table 1). The transition costs included the costs related to training staff and the time necessary for planning, coordination, informatics and workflow revision, and quality improvement, and start-up equipment purchase, and interoperable EHR software expenses (EHR software development cost for the first year and monthly lease fees for the subsequent years) [26]. Recurring costs included salaries for a general dental practitioner (1 full-time equivalent (FTE)), dental hygienists (1.4 FTE), and chairside assistants (1.5 FTE), and the costs associated with delivering dental services, such as dental supplies and drugs. These estimates were calculated from the fact that average general dental practitioners hire dental hygienists and chairside assistants 77.5% and 86.3% of the time, and average numbers of dental hygienists and chairside assistants per dentist among those who employ these staff are 1.8 and 1.7, respectively [34].

### 2.5. Primary and Secondary Outcome Metrics

The primary outcome was changes in net revenue per integrated practice per year. We computed the main outcome metric as the total reimbursements for dental services minus the total cost of service provision. Our secondary outcome metrics included (1) costs of dental service integration and (2) gross revenues for dental service integration. The primary and secondary outcomes were computed per annum for both the first and subsequent years. 

### 2.6. Sensitivity Analyses

In an integrated setting, an increase in dental service utilization is expected due to theoretically easier access to dental care. Moreover, with recent findings on association between periodontal diseases and chronic conditions, a number of insurance companies have started offering 100% coverage for nonsurgical periodontal treatment to those with chronic conditions, such as diabetes, cardiovascular diseases, rheumatoid arthritis, and HIV/AIDS, which may increase utilization of periodontal treatment services [35,36,37]. The average hours per day a general dental practitioner spends in the dental office is 6.3, and 26.5% of surveyed general dentists perceived their workload to be “not busy enough” [38]. In order to estimate expected changes in net revenue from changes in utilization rates, we simulated potential increases or decreases in utilization rates in all procedure types from 50% (7 patients/day) to 120% (17 patients/day, dental practitioners spending time in the dental office for a maximum 7.6 hours per day) of baseline values. 

Next, based on findings from one of the demonstration projects [17], we assessed how increases in preventive care utilization (radiographs, prophylaxis, fluoride varnish application, and sealant placement) would result in changes in net revenue. Because preventive care can be performed by hygienists, we simulated changes in net revenue from employing an additional hygienist to accommodate potential increases in preventive dental care. The number of patients a dental hygienist could accept was capped at the current average number of hygienist appointments at general dental practices nationwide [38]. We evaluated the impact of varying rates of increase in preventive care utilization on total net revenue with an additional dental hygienist. 

Lastly, we simulated different payer distributions across the patient visits. In the base-case scenario, we used the national average payer distribution for medical and dental practices; 66% private, 25% public, 8 % uninsured for medical, and 52% private, 19% public, and 29% uninsured for dental practices (in dental practices, we did not include Medicare as public as dental benefits are not covered under Medicare with the exception of select Medicare Advantage plans). In this sensitivity analysis, we evaluated the impact of different patient payer distributions in certain settings. Community Health Centers (CHCs) serve a higher percentage of publicly insured or uninsured patients than the national average: 17% private, 59% public (49% Medicaid), and 24% uninsured [39]. In order to account for the fact that most patients seen by the dentist will come from the primary care practice after integration, we simulated average payer distribution at primary care practices: 45% private, 48 % public (17% Medicaid), and 7% uninsured [40]. In these scenarios, we assumed that same proportions of privately insured and Medicare patients have private dental insurance as in the base case, and calculated estimated dental insurance payer distributions for each setting.

## 3. Results

### 3.1. Base-Case Analyses

Among the fifteen procedure types that were determined to be routinely delivered by general dental practitioners, diagnostic examination and cleaning (prophylaxis) had the highest utilization rates, followed by radiographs (Supplementary Figure S2). The privately insured population visited dental practices for routine check-ups and cleanings at a higher rate than publicly insured or uninsured populations. While 62.2% (95% CI: 61.0, 63.3) of the total dental visits in a given year were for examinations in the privately insured population, publicly insured and uninsured populations visited a dental practice for examinations 57.6% (95% CI: 55.6, 59.5) and 53% (95% CI: 48.2, 58.3) of the time, respectively. However, the rate of tooth extraction was more than twice as high among publicly insured and uninsured patients, which might be due to less-frequent routine dental visits. Uninsured patients visited a dental practice for tooth extraction 21.6% (95% CI: 19.6, 23.7) of the time, whereas privately and publicly insured patients visited a dental practice for tooth extraction 5.8% (95% CI: 5.6, 6.0) and 14.4% (95% CI: 13.8, 15.0) of the time, respectively. 

When dental services by a general dental practitioner were offered in the simulated integrated care setting, the primary outcome of net revenue was positive after the first year of integration. Due to transition costs and start-up expenses, the net revenue in the first year of integration was negative, -$92,053 (95% CI: −93,054, −91,052) (Table 2). After the first year, annual net revenue for the subsequent years was $104,316 (95% CI: 103,315, 105,316) per practice after the first year, assuming the same utilization rates as existing patients who completed dental visits. 

**Figure 2:**
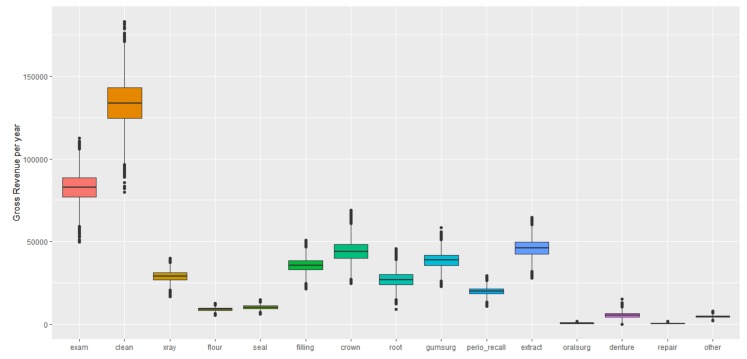
Gross revenue by procedure type, showing the minimum (lower whisker), maximum (upper whisker), median (center of the box), lower quartile (bottom of box), and upper quartile (top of box) values. Exam = diagnostic; clean: prophylaxis; X-ray = radiographic image; flour = fluoride; seal = sealant; root = root canal; gumsurg = periodontal scaling, root planning or gum; extract = extraction/ tooth pulled; repair = repair of bridges/dentures or relining.

The total gross revenue from dental practices was $493,830 (95% CI: 492,831, 494,828). The highest-revenue-generating procedure type was cleanings, with a gross revenue of $130,350 (95% CI: 130,088, 130,612), followed by diagnostic examinations and extractions, with gross annual revenues of $80,910 (95% CI: 80,747, 81,072) and $53,693 (95% CI: 53,574, 53,811), respectively (Figure 2). The least-revenue-generating procedure type was repair, such as repairing or rebasing dentures, resulting in gross annual revenue of $512 (95% CI: 508, 518).

### 3.2. Sensitivity Analyses

When overall utilization rates varied from half to twice their baseline values, net revenue per annum after the first year of integration remained positive as long as the overall utilization rates decreased by less than 25% (Table 2). Because of a greater number of adults visiting a physician annually than a dental practitioner and increased rates of enhanced dental benefits among patients with chronic conditions who are more likely to have more frequent medical visits, we expect that medical–dental integration would increase access to and utilization of dental care. When the modeled utilization rates were increased by 20%, net revenue per annum was $187,191 (95% CI: 186,052, 188,330).

Next, we evaluated the impact of hiring an additional dental hygienist to perform four types of procedures (radiographs, prophylaxis, fluoride varnish application, and sealant placement) to accommodate potential increases in preventive dental care with integration. When preventive care utilization increased by more than 53%, hiring an additional full-time dental hygienist resulted in a higher net revenue. If a full-time dental hygienist is hired and works at full capacity (performing diagnostic and preventive procedures at approximately the same rates as the average dental hygienist currently seeing patients in the U.S.), the expected net revenue was $169,208 (95% CI: 168,071, 170,345), which was a 62.2% increase from before employing the additional dental hygienist (Table 2). 

**Figure 3:**
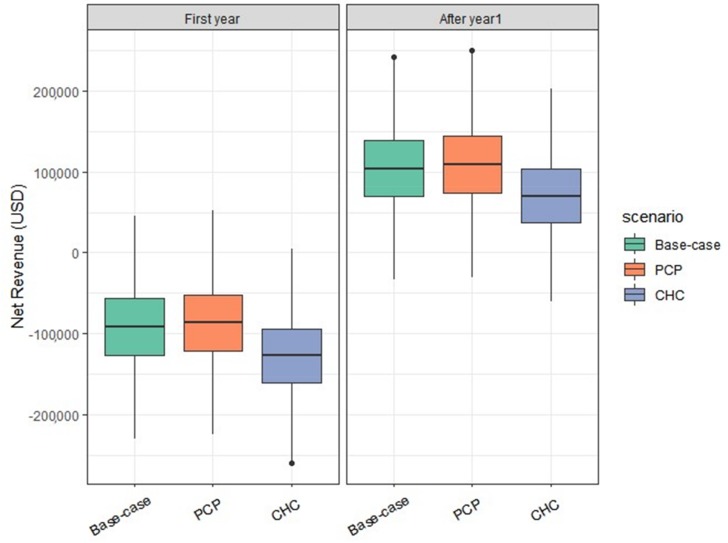
Impact of different payer distributions, showing the minimum (lower whisker), maximum (upper whisker), median (center of the box), lower quartile (bottom of box), and upper quartile (top of box) values. Base case = national average; PCP = primary care practice; CHC = community health center.

When we simulated payer distributions at a CHC with a high proportion of publicly insured, the expected net revenue was $70,099 (95% CI: 69,136, 71,061), $34,217 lower than the net revenue from the base-case scenario, due primarily to lower reimbursement rates from public payers and the types of dental procedures these patients receive (Figure 3 and Supplementary Table S5). In the average primary care provider setting, the net revenue was $108,764 (95% CI: 107,744, 109,783), which was $4448 higher than the net revenue from the base-case scenario. 

## 4. Discussion

With increased interest in the potential for integrated medical–dental care, our study evaluated the financial viability of primary integrated services—primary medicine and primary dentistry—to achieve whole-person care. We found that the net revenue changes after the first year of integration would remain positive when the integrated care could maintain at least 75% of current patient volume and the payer distribution. Serving a high proportion of patients covered by public dental insurance would result in a lower net revenue due to lower reimbursement rates. With the potential increase in utilization of basic preventive services due to integration, employing an additional hygienist to accommodate increased demand would increase the net revenue up to 62% if the hygienist worked at full capacity.

A key obstacle to successful integration of medical and dental service provision has been the substantial infrastructural investments required, such as interoperable EHRs, shared or commonly managed facilities, and a multidisciplinary workforce. While an interoperable EHR promotes well-informed care and treatment planning as well as coordination of the scheduling and billing of patient visits, it is relatively new concept and involves technical hurdles [41]. In our study, we implemented a monthly leased software option, a reasonably integrated option; however, it could be home grown with greater financial investment. For this and other reasons, the integration of medical and dental services can be a highly resource-intensive model to implement. 

Our results suggest that facilities would experience negative net revenue from implementation in the first year; however, the net revenue for successful implementation would remain positive. While our study was limited to evaluating the financial viability of the integrated care, the expected benefits from this integration may extend beyond positive revenue. Integrated care facilitates timely delivery of diagnostic, preventive, and treatment services to improve patient health and reduce inefficiency in care delivery. Integrated care with dental, psychiatric, and allied health service has been also supported in other countries [42], and due to significant overlap in training between dental and medical students in many European countries, it is practically viable outside the U.S. [43]. Based on recent findings on the association between oral health status and chronic conditions [3,4,5] and potential cost savings from co-management of these diseases [44,45], integrated medical–dental practice could be expected to improve health outcomes of the population and result in cost savings in the overall healthcare expenditures in the U.S. 

Our analysis has limitations inherent to simulation modeling based on secondary data sources. First, we simulated the utilization and cost of dental services at procedure level based on claims data from a mostly privately insured population. Although we extrapolated from nationally representative survey data to make projections about publicly insured and uninsured populations, some information loss is to be expected by grouping a number of procedure codes into different categories. An additional logical step for future research is to gain access to claims data from publicly insured populations, such as Center for Medicare and Medicaid Services (CMS) data, to identify whether incorporating procedure-level data in this population would alter the findings of our study [46]. Furthermore, we lacked sufficiently rigorous data to expand our model to incorporate regional variation in service utilization and payer distribution, such as urban vs. rural or by state. Dentist supply, dental care demand, and payer distributions vary a lot across geographic location. While our study results are based on national averages, medical–dental integration would likely yield higher revenue in one setting than the in other. In the absence of robust data about how much patient volume would change in terms of dental service need, we did not make any assumptions about the trends in dental utilization or payer distribution of the population over time. Moreover, we assumed that only a subset of dental procedures would be performed by general dental practitioners at an integrated setting under a fee-for-service scenario, and specialty services would be referred out. However, it may not be applicable to CHC where it accepts encounter-based payment, and there is a possibility that some CHCs may provide specialty services that are not covered, which would alter the financial impact of the integrated care practice. Finally, the proprietary nature of the ADA data used here is a limitation for broad usage; the potential availability of other practice cost registries or data from a strong medical–dental integrated practice may eventually lead to the wider availability of financial data for practice planning, and it remains as an area for future research. 

## 5. Conclusions

Our findings suggest that medical–dental integration is financially viable. Given that more adults visit a physician than a dentist annually and that in some case enhanced dental benefits are being offered to patients with chronic conditions, medical–dental integration could improve patient health and reduce inefficiency in care delivery. Furthermore, it has potential value to provide comprehensive whole-person care through bidirectional referrals and sharing patient information, which would provide a critical opportunity to bridge the gap between dentistry and medicine.

## Figures and Tables

**Table 1:** Input data for the dental care integration model. Data are expressed as mean (SD).

Parameters	Value	Source
Practice/patient characteristics		
Number of patient visits per dentist (including hygienist appointment) per year	3415 (347)	ADA HPI [21]
Number of patient visits per dentist (excluding hygienist appointment) per year	1831 (127)	ADA HPI [21]
Number of patient visits per hour	2.3	ADA HPI [21]
Number of hours spent on patient visits per day	6.1	ADA HP I[21]
Health insurance payer distribution of overall population (proportion with dental insurance in each group)		MEPS [22]
Private	0.66 (0.01) [0.69 (0.01)]	
Public	0.25 (0.01) [0.02 (0.01)]	
Uninsured	0.08 (0.01) [0.04 (0.01)]	
Dental insurance payer distribution		MEPS [22]
Private	0.52 (0.05)	
Public	0.19 (0.03)	
Uninsured	0.29 (0.01)	
Utilization rates		
CDT procedure level utilization rate (privately insured)	Supplementary Table S1	Aetna Warehouse
Relative scales of utilization rates (public and uninsured)	Supplementary Table S1	MEPS [22]
Costs of dental procedures		
CDT procedure level costs (privately insured)	Supplementary Table S2	Aetna Warehouse
Reimbursement rates relative to private insurance	Supplementary Table S3	MEPS [22]
Expenses		
Dentist salary	152,210 (20,830)	ADA HPI [21]
Hygienist	74,070 (12,680)	Bureau of Labor Statistics [23]
Chairside assistant	37,630 (6870)	Bureau of Labor Statistics [23]
Primary care physician (hourly)	$98 (7)	MGMA [24]
Medical Assistant (hourly)	$15.1(2)	Bureau of Labor Statistics [23]
Recurring costs		
Clinical space	$1014 (290)	MGMA [24]
Dental supplies	6.4% of gross billing	ADA [25]
Drugs	0.3% of gross billing	ADA [25]
Dental lab charges	6.4% of gross billing	ADA [25]
Repairs of dental equipment	0.7% of gross billing	ADA [25]
Annual depreciation cost on dental equipment	2.2% of gross billing	ADA [25]
EHR software monthly fee	$135 (25)	Delta Dental [26]
Transition Costs (applied to the first year)		
Equipment, computers, software	$195,000 (2000)	ADA [27]
Integrated EHR development	$5000	Delta Dental [26]
Planning, coordination, informatics and workflow revision, and quality improvement during setup period	$1411 (73)	Prior pilot projects in other disciplines [28,29]

ADA = American Dental Association; HPI = Health Policy Institute; MGMA = Medical Group Management Association; EHR = electronic health records.

**Table 2:** Costs and revenues from medical–dental integration, per practice per year.

	Cost, Year 1 (USD)	Cost, after Year 1 (USD)	Gross Revenue (USD)	Net Revenue, Year 1 (USD)	Net Revenue, After Year 1 (USD)
Base case	585,927 (585,335, 586,519)	389,514 (388,923, 390,104)	493,830 (492,831, 494,828)	−92,053 (−93,054, −91,052)	104,316 (103,315, 105,316)
Overall utilization (patient visit volume) change					
50%	546,758 (546,184, 547,331)	350,372 (349,799, 350,944)	247,654 (247,148, 248,160)	−299,227 (−299,929, −298,526)	−102,717 (−103,416, −102,019)
60%	554,582 (554,006, 555,158)	358,180 (357,604, 358,755)	296,759 (296,157, 297,362)	−257,842 (−258,595, −257,089)	−61,420 (−62,170, −60,669)
70%	562,408 (562,408, 561,829)	366,018 (365,439, 366,596)	346,057 (345,354, 346,760)	−216,448 (−217,256, −215,639)	−19,960 (−20,768, −19,152)
80%	570,238 (569,655, 570,821)	373,822 (373,240, 374,404)	395,141 (394,341, 395,940)	−175,034 (−175,904, −174,164)	21,318 (20,450, 22,186)
90%	578,076 (577,489, 578,663)	381,689 (381,103, 382,275)	444,617 (443,719, 445,516)	−133,575 (−134,507, −132,644)	62,928 (61,994, 63,862)
110%	593,784 (593,188, 594,381)	397,350 (396,777, 397,966)	543,252 (542,160, 544,344)	−50,490 (−51,557, −49,421)	145,880 (144,812, 146,948)
120%	601,601 (601,000, 602,202)	405,067 (404,582, 405,782)	592,374 (591,183, 593,564)	−9145 (−10,287, −8004)	187,191 (186,052, 188,330)
Preventive service utilization change with additional dental hygienist					
50% increase	673,080 (672,304, 673,857)	476,603 (475,843, 477,363)	576,377 (575,362, 577,391)	−96,703 (−97,787, −95,620)	99,774 (98,657, 100,889)
60% increase	675,706 (674,927, 676,484)	479,228 (478,469, 479,988)	592,887 (591,868, 593,907)	−82,818 (−83,897, −81,738)	113,659 (112,539, 114,778)
70% increase	678,331 (677,550, 679,112)	481,854 (481,094, 482,613)	609,399 (608,373, 610,425)	−68,932 (−70,008, −67,856)	127,545 (126,421, 128,669)
80% increase	680,955 (680,171, 681,738)	484,477 (483,717, 485,237)	625,899 (624,868, 626,931)	−55,055 (−56,127, −53,982)	141,422 (140,294, 142,550)
90% increase	683,580 (682,795, 684,366)	487,103 (486,343, 487,863)	642,413 (64,1374, 643,452)	−41,167 (−42,236, −40,097)	155,310 (154,178, 156,442)
100% increase(full capacity)	686,208 (685,420, 686,995)	489,730 (488,970, 490,491)	658,939 (657,894, 659,985)	−27,268 (−28,335, −26,201)	169,208 (168,071, 170,345)

## Supplementary Materials

The following are available online at https://www.mdpi.com/1660-4601/17/6/2154/s1, Table S1: Utilization rates by procedure type and by insurance, Table S2: Costs by dental procedure types (commercial insurance), Table S3: Reimbursed rates by insurance status, Table S4: Procedure codes offered by general dentist and MEPS dental practice category matching, Table S5: Sensitivity analysis results—different payer distributions, Figure S1: Payment source distribution by insurance status, Figure S2: Utilization rates by procedure and insurance types.
