# Supplementary material for: Dental Practice Integration Into Primary Care: A Microsimulation of Financial Implications for Practices
Source: Int J Environ Res Public Health. 2020 Mar 24;17(6):0. doi: 10.3390/ijerph17062154 (PMC7175120; doi:10.3390/ijerph17062154)
Supplement: Supplementary file 1 [file ijerph-17-02154-s001.pdf]

Dental Practice Integration into Primary Care: A Microsimulation of Financial Implications for Practices

Appendix

Table A1. Utilization rates by procedure type and by insurance

Table A2. Costs by dental procedure types (private insurance)

Table A3. Reimbursed rates by insurance status

Table A4. Procedure codes offered by general dentist and MEPS dental practice category matching

Table A5. Sensitivity analysis results – different payer distributions

Figure A1. Payment source distribution by insurance status

Figure A2. Utilization rates by procedure and insurance types

Table A1. Utilization rates by procedure type and by insurance

Privately insured population

Type	Mean	Lower	Upper
d_exam	0.621811	0.005983	0.011727
d_clean	0.631954	0.005987	0.011735
d_xray	0.278718	0.004602	0.009019
d_flour	0.161022	0.003537	0.006932
d_seal	0.086595	0.000905	0.001773
d_filling	0.068891	0.001788	0.003504
d_crown	0.030781	0.001387	0.002719
d_root	0.012865	0.00191	0.003744
d_gumsurg	0.085884	0.000773	0.001516
d_perio_recall	0.086635	0.000677	0.001328
d_extract	0.058335	0.001046	0.00205
d_oralsurg	0.001174	0.000392	0.000768
d_denture	0.001566	0.000657	0.001287
d_repair	0.000786	0.000373	0.00073
d_other	0.017256	0.000769	0.001507

Publicly insured population

Type	Mean	Lower	Upper
d_exam	0.575843	0.010008	0.019615
d_clean	0.565468	0.009859	0.019324
d_xray	0.266431	0.00722	0.01415
d_flour	0.174528	0.004676	0.009164
d_seal	0.167018	0.001966	0.003853
d_filling	0.086353	0.002972	0.005826
d_crown	0.02386	0.002022	0.003962
d_root	0.007957	0.001992	0.003904
d_gumsurg	0.063327	0.001044	0.002046
d_perio_recall	0.046878	0.000491	0.000963
d_extract	0.14418	0.002903	0.00569
d_oralsurg	0.001149	0.000723	0.001417
d_denture	0.00552	0.0024	0.004704
d_repair	0.001575	0.000901	0.001766
d_other	0.013562	0.001106	0.002167

Uninsured population

Type	Mean	Lower	Upper
d_exam	0.53257	0.02567	0.050314
d_clean	0.521235	0.024712	0.048435
d_xray	0.265914	0.019289	0.037806
d_flour	0.09359	0.009214	0.018059
d_seal	0.11003	0.003058	0.005993
d_filling	0.108129	0.011444	0.02243
d_crown	0.03655	0.006812	0.013351
d_root	0.032449	0.008703	0.017058
d_gumsurg	0.066516	0.003261	0.006392
d_perio_recall	0.062916	0.001218	0.002387
d_extract	0.216713	0.010164	0.019922
d_oralsurg	0.001682	0.002438	0.004778
d_denture	0.003531	0.003559	0.006975
d_repair	0.001614	0.00213	0.004174
d_other	0.02172	0.004563	0.008943

Table A2. Costs by dental procedure types (private insurance)

Procedure type	Mean	SD
d_exam	42.48	14.8
d_clean	68.38	8.6
d_xray	32.37	7.3
d_flour	18.52	5.4
d_seal	30.68	14.0
d_filling	138.95	31.3
d_crown	440.34	82.1
d_root	525.43	54.6
d_gumsurg	150.96	41.9
d_perio_recall	80.22	14.9
d_extract	139.77	44.7
d_oralsurg	117.26	63.5
d_denture	644.53	205.1
d_repair	128.47	55.6
d_other	81.78	61.1

Table A3. Reimbursed rates (calculated as total expenditure / total charged)

	private		public		uninsured	
	mean	se	mean	se	mean	se
d_exam	0.888	0.004	0.701	0.007	0.828	0.044
d_clean	0.892	0.005	0.706	0.007	0.886	0.018
d_xray	0.890	0.005	0.703	0.007	0.876	0.021
d_flour	0.896	0.009	0.612	0.006	0.819	0.051
d_seal	0.920	0.016	0.604	0.011	0.741	0.104
d_filling	0.888	0.006	0.692	0.010	0.876	0.035
d_crown	0.891	0.011	0.817	0.018	0.811	0.071
d_root	0.873	0.015	0.715	0.039	0.943	0.035
d_gumsurg	0.930	0.017	0.838	0.046	0.662	0.150
d_perio_recall	0.934	0.022	0.550	0.123	0.952	0.035
d_extract	0.895	0.010	0.693	0.011	0.789	0.128
d_oralsurg	0.836	0.031	0.657	0.040	0.947	0.060
d_denture	0.805	0.034	0.747	0.029	0.901	0.061
d_repair	0.815	0.057	0.733	0.060	0.517	0.168
d_other	0.860	0.021	0.734	0.042	0.788	0.085

Table A4. Procedure codes offered by general dentist and MEPS dental practice category matching

MEPS Category	Procedure code	Description of Service
d_exam	D0120	Periodic oral evaluation — established patient
d_exam	D0140	Limited oral evaluation - problem focused
d_exam	D0145	Oral evaluation for a patient under three years of age and counseling with primary caregiver
d_exam	D0150	Comprehensive oral evaluation — new established patient
d_exam	D0160	Detailed and extensive oral evaluation —problem focused, by report
d_exam	D0180	Comprehensive periodontal evaluation —new or established patient
d_xray	D0210	Intraoral - complete series of radiographic images
d_xray	D0220	Intraoral - periapical first radiographic image
d_xray	D0230	Intraoral - periapical each additional radiographic image
d_xray	D0272	Bitewings - two radiographic images
d_xray	D0273	Bitewings - three radiographic images
d_xray	D0274	Bitewings - four radiographic images
d_xray	D0277	Vertical bitewings - 7 to 8 radiographic images
d_clean	D1110	Prophylaxis - adult
d_clean	D1120	Prophylaxis - child
d_flour	D1206	Topical application of fluoride varnish
d_flour	D1208	Topical application of fluoride – excluding varnish
d_seal	D1351	Sealant - per tooth
d_seal	D1352	Preventive resin restoration in a moderate to high caries risk patient — permanent tooth
d_filling	D2140	Amalgam - one surface, primary or permanent
d_filling	D2150	Amalgam - two surfaces, primary or permanent
d_filling	D2160	Amalgam - three surfaces, primary or permanent
d_filling	D2161	Amalgam - four or more surfaces, primary or permanent
d_filling	D2330	Resin-based composite - one surface, anterior
d_filling	D2331	Resin-based composite - two surfaces, anterior
d_filling	D2332	Resin-based composite - three surfaces, anterior
d_filling	D2335	Resin-based composite - four or more surfaces or involving incisal angle (anterior)
d_filling	D2390	Resin-based composite crown, anterior
d_filling	D2391	Resin-based composite - one surface, posterior
d_filling	D2392	Resin-based composite - two surfaces,posterior
d_filling	D2393	Resin-based composite - three surfaces, posterior
d_filling	D2394	Resin-based composite - four or more surfaces, posterior
d_crown	D2710	Crown - resin-based composite
d_crown	D2720	Crown - resin with high noble metal
d_crown	D2721	Crown - resin with predominantly base metal
d_crown	D2722	Crown - resin with noble metal
d_crown	D2740	Crown - porcelain/ceramic substrate
d_crown	D2750	Crown - porcelain fused to high noble metal
d_crown	D2751	Crown - porcelain fused to predominantly
d_crown	D2752	Crown - porcelain fused to noble metal
d_crown	D2780	Crown - ¾ cast high noble metal
d_crown	D2783	Crown - ¾ porcelain/ceramic
d_crown	D2790	Crown - full cast high noble metal

d_crown	D2794	Crown - titanium
d_crown	D2910	Re-cement inlay
d_crown	D2920	Re-cement or re-bond crown
d_crown	D2929	Prefabricated porcelain/ceramic crown - primary tooth
d_crown	D2930	Prefabricated stainless steel crown - primary tooth
d_crown	D2931	Prefabricated stainless steel crown - permanent tooth
d_crown	D2940	Protective restoration
d_crown	D2950	Core buildup, including any pins when required
d_crown	D2951	Pin retension - per tooth
d_crown	D2952	Post and core in addition to crown, indirectly fabricated
d_crown	D2954	Prefabricated post and core in addition to crown
d_crown	D2961	Labial veneer (resin laminate) - laboratory
d_crown	D2962	Labial veneer (porcelain laminate) - laboratory
d_crown	D2980	Crown repair necessitated by restorative material failure
d_root	D3110	Pulp cap - direct (excluding final restoration)
d_root	D3120	Pulp cap - indirect (excluding final restoration)
d_root	D3220	Therapeutic pulpotomy (excluding final restoration)…
d_root	D3221	Pulpal debridement, primary and permanent teeth
d_root	D3230	Pulpal therapy (resorbable filling) - anterior, primary tooth (excluding final restoration)
d_root	D3240	Pulpal therapy (resorbable filling) - posterior, primary tooth (excluding final restoration)
d_root	D3310	Endodontic therapy, anterior tooth (excluding final restoration)
d_root	D3320	Endodontic therapy, bicuspid tooth (excluding final restoration)
d_root	D3330	Endodontic therapy, molar (excluding final restoration)
d_gumsurg	D4320	Provisional splinting - intra
d_gumsurg	D4321	Provisional splinting - extracoronal
d_gumsurg	D4341	Periodontal scaling and root planing - four or more teeth per quadrant
d_gumsurg	D4342	Periodontal scaling and root planing - one to three teeth per quadrant
d_gumsurg	D4355	Full mouth debridement to enable comprehensive evaluation and diagnosis
d_perio_recall	D4910	Periodontal maintenance
d_dentures	D5110	Complete denture - maxillary
d_dentures	D5120	Complete denture - mandibular
d_dentures	D5130	Immediate denture - maxillary
d_dentures	D5140	Immediate denture - mandibular
d_dentures	D5211	Maxillary partial denture - resin base (including any conventional clasps, rests and teeth)
d_dentures	D5212	Mandibular partial denture - resin base (including any conventional clasps, rests and teeth
d_dentures	D5213	Maxillary partial denture - cast metal framework with resin denture bases and teeth)
d_dentures	D5214	Mandibular partial denture - cast metal framework with resin denture bases (including any conventional clasps, rests, and teeth)
d_dentures	D5221	Immediate maxillary partial denture - resin base (including any conventional clasps, rests and teeth
d_dentures	D5222	Immediate mandibular partial denture - resin base (including any conventional clasps, rests and teeth
d_dentures	D5223	Immediate maxillary partial denture - cast metal framework with resin denture bases (including any conventional clasps, rests and teeth)

d_dentures	D5224	Immediate mandibular partial denture - cast metal framework with resin denture bases (including any conventional clasps, rests and teeth)
d_dentures	D5225	Maxillary partial denture — flexible base (including any clasps, rests, and teeth)
d_dentures	D5226	Mandibular partial denture - flexible base (including any clasps, rests, and teeth)
d_repair	D5510	Repair broken complete denture base
d_repair	D5520	Replace missing or broken teeth — complete denture (each tooth)
d_repair	D5610	Repair resin denture base
d_repair	D5630	repair or replace broken clasp
d_repair	D5640	Replace broken teeth - per tooth
d_repair	D5650	Add tooth to existing partial denture
d_repair	D5660	Add clasp to existing partial denture - per tooth
d_repair	D5710	Rebase complete maxillary denture
d_repair	D5711	Rebase complete mandibular denture
d_repair	D5720	Rebase maxillary partial denture
d_repair	D5721	Rebase mandibular partial denture
d_repair	D5730	Reline complete maxillary denture(chairside)
d_repair	D5731	Reline complete mandibular denture (chairside)
d_repair	D5740	Reline maxillary partial
d_repair	D5741	reline mandibular partial
d_repair	D5750	Reline complete maxillary denture (laboratory)
d_repair	D5751	Reline complete mandibular denture (laboratory)
d_repair	D5760	reline maxillary partial
d_repair	D5761	reline mandibular partial
d_extract	D7111	Extraction, coronal remnants -deciduous tooth
d_extract	D7140	Extraction, erupted tooth or exposed root (elevation and/or forceps removal)
d_extract	D7210	Surgical removal of erupted tooth requiring removal of bone and/or sectioning of tooth, and including elevation of mucoperiosteal flap if indicated
d_extract	D7230	Removal of impacted tooth -partially bony
d_extract	D7240	Removal of impacted tooth -completely bony
d_extract	D7250	Surgical removal of residual tooth roots (cutting procedure)
d_extract	D7251	Coronectomy - intentional partial tooth removal
d_oralsurg	D7286	Incisional biopsy of oral tissue - soft
d_oralsurg	D7310	Alveoloplasty in conjunction with extractions - four or more teeth or tooth spaces, per quadrant
d_oralsurg	D7970	Excision of hyperplastic tissue - per arch
d_other	D9110	Palliative (emergency) treatment of dental pain - minor procedure
d_other	D9230	Inhalation of nitrous oxide/analgesia, anxiolysis
d_other	D9310	Consultation - diagnostic service provided by dentist or physician other than requesting dentist or physician
d_other	D9910	Application of desensitizing medicament
d_other	D9940	Occlusal guard, by report
d_other	D9941	Fabrication of athletic mouth guard
d_other	D9951	Occlusal adjustment - limited
d_other	D9952	Occlusal adjustment - complete

Table A5. Costs and revenues from medical-dental integration, per practice per year

Parameters	Cost, year 1 (USD)	Cost, after year 1 (USD)	Gross revenue (USD)	Net revenue, year 1 (USD)	Net revenue, after year 1 (USD)
Base case	585,927 (585,335, 586,519)	389,514 (388,923, 390,104)	493,830 (492,831, 494,828)	-92,053 (-93,054, -91,052)	104,316 (103,315, 105,316)
Different payer distributions (dental insurance coverage distributions)					
Community health center (18% private, 50% public, 32% uninsured)	586,347 (585,763, 586,930)	389,006 (388,421, 389,590)	459,105 (458,174, 460,035)	-127,238 (-128,186, -126,289)	70,099 (69,136, 71,061)
Primary care provider (50% private, 17% public, 33% uninsured)	585,682 (586,268, 585,095)	389,970 (389,393, 390,546)	498,733 (497,726, 499,740)	-87,319 (-88,324, -86,313)	108,764 (107,744, 109,783)

Figure A1. Payment source distribution by insurance type

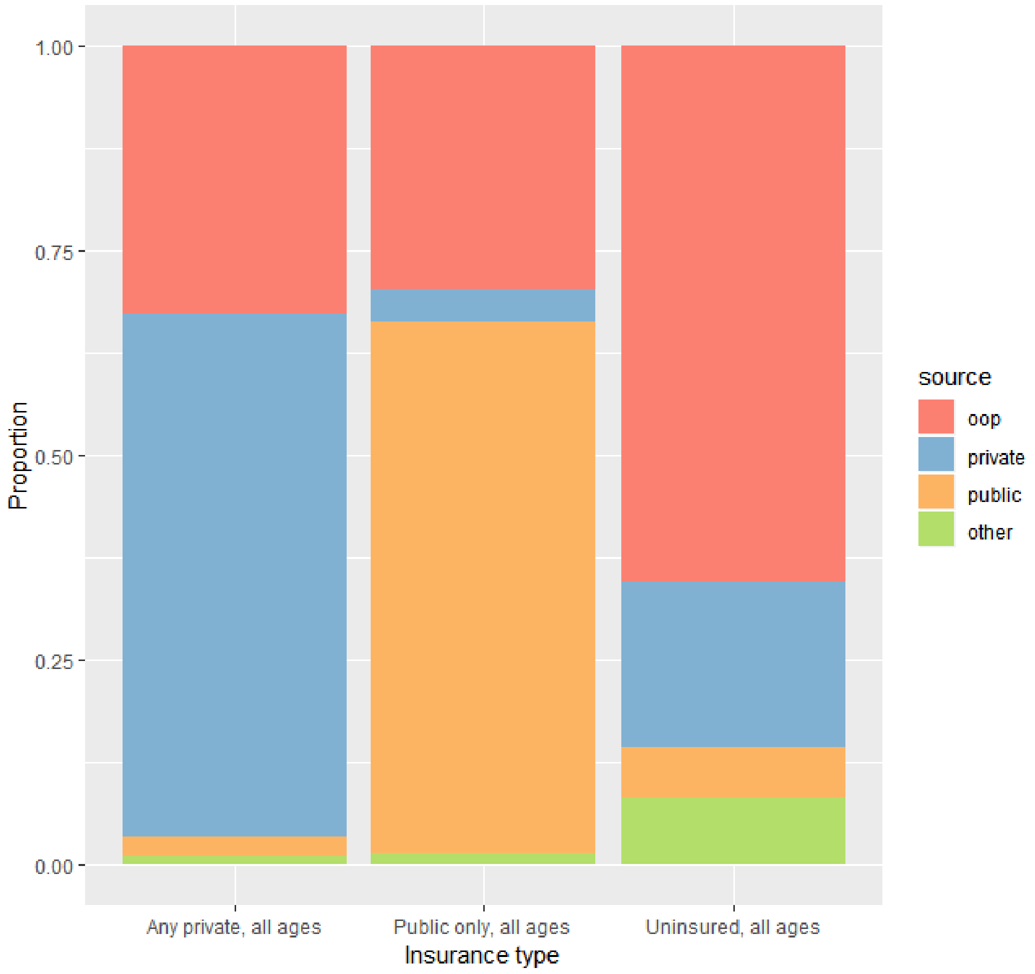

oop: out-of-pocket;

other: Tricare, Veterans Affairs, Other state, Other federal, workers compensation, other unclassified

Figure A2. Utilization rates by procedure and insurance types

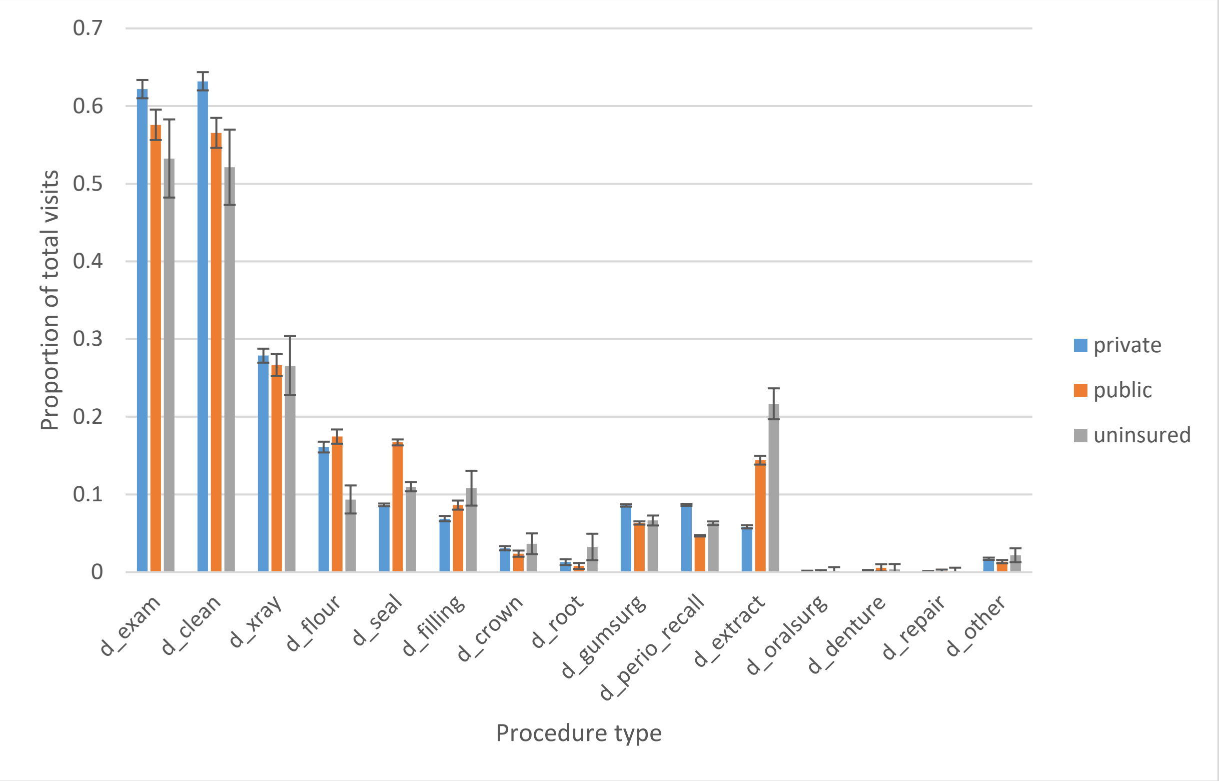

exam: diagnostic; clean: prophylaxis; xray: radiographic image; flour: fluoride; seal: sealant; root: root canal; gumsurg: periodontal scaling, root planning or gum; extract: extraction/ tooth pulled; repair: repair of bridges/dentures or relining
